# Brain Morphometric Alterations in Focal to Bilateral Tonic–Clonic Seizures in Epilepsy Associated With Excitatory/Inhibitory Imbalance

**DOI:** 10.1111/cns.70129

**Published:** 2024-11-24

**Authors:** Qiuxing Lin, Wei Li, Yingying Zhang, Yuming Li, Peiwen Liu, Xiang Huang, Kailing Huang, Danyang Cao, Qiyong Gong, Dong Zhou, Dongmei An

**Affiliations:** ^1^ Department of Neurology, West China Hospital Sichuan University Chengdu Sichuan China; ^2^ Department of Radiology, Huaxi MR Research Center, West China Hospital Sichuan University Chengdu Sichuan China

**Keywords:** Allen Human Brain Atlas, excitatory/inhibitory balance, focal to bilateral tonic–clonic seizures, imaging transcriptome analysis, morphometric alterations

## Abstract

**Background:**

Focal to bilateral tonic–clonic seizures (FBTCS) represent the most severe seizure type in temporal lobe epilepsy (TLE), associated with extensive network abnormalities. Nevertheless, the genetic and cellular factors predispose specific TLE patients to FBTCS remain poorly understood. This study aimed to elucidate the relationship between brain morphometric alterations and transcriptional profiles in TLE patients with FBTCS (FBTCS+) compared to those without FBTCS (FBTCS−).

**Methods:**

We enrolled 126 unilateral TLE patients (89 FBTCS+ and 37 FBTCS−) along with 60 age‐ and gender‐matched healthy controls (HC). We assessed gray matter volume to identify morphometric differences between patients and HC. Partial least squares regression was employed to investigate the association between the morphometric disparities and human brain transcriptomic data obtained from the Allen Human Brain Atlas.

**Results:**

Compared with HC, FBTCS+ patients exhibited morphometric alterations in bilateral cortical and subcortical regions. Conversely, FBTCS− patients exhibited more localized alterations. Imaging transcriptomic analysis revealed both FBTCS− and FBTCS+ groups harbored genes that spatially correlated with morphometric alterations. Additionally, pathway enrichment analysis identified common pathways involved in neural development and synaptic function in both groups. The FBTCS− group displayed unique pathway enrichment in catabolic processes. Furthermore, mapping these genes to specific cell types indicated enrichment in excitatory and inhibitory neurons in the FBTCS− group, while FBTCS+ group only enriched in excitatory neurons. The distinct cellular expression differences between FBTCS− and FBTCS+ groups are consistent with the distribution patterns of GABAergic expression.

**Conclusion:**

We applied imaging transcriptomic analysis linking the morphometric changes and neurobiology in TLE patients with and without FBTCS, including gene expression, biological pathways, cell types, and neurotransmitter receptors. Our findings revealed abnormalities in inhibitory neurons and altered distribution patterns of GABAergic receptors in FBTCS+, suggesting that an excitatory/inhibitory imbalance may contribute to the increased susceptibility of certain individuals to FBTCS.

## Graphical abstract

Schematic diagram of methodology. (A) T1‐weighted image of each subject was parcellated into 322 cortical and subcortical regions, and gray matter volume of each region was computed by Freesurfer. Then regional case–control differences were estimated. (B) Gene expression profiles of each region (left hemisphere only) were extracted from AHBA database and obtained 159 × 15,633 regional gene matrix. (C) PLS regression was applied to link case–control *t*‐values and gene expression data. Then, pathway enrichment analysis and cell type assignment were conducted on the gene list of first component of PLS to identify different biological pathway and cell types between groups. Additionally, the GABAergic atlas was used to explore the relationship between inhibitory neurotransmitter and morphometric alterations. AHBA, Allen Human Brain Atlas; PLS, partial least squares.

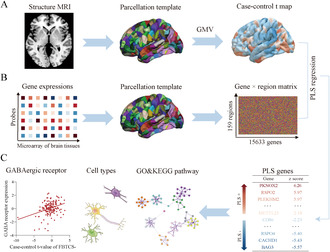

## 1 Introduction

Temporal lobe epilepsy (TLE) stands as the most common type of focal epilepsy in adults, characterized as a polygenic and complex neurological disorder [1]. Focal to bilateral tonic–clonic seizures (FBTCS), formerly known as secondarily generalized tonic–clonic seizures, is the most severe seizure type in TLE due to the comprehensive loss of body control and a heightened risk of sudden unexpected death [2, 3], alongside a lesser responsiveness to medical treatment or epilepsy surgery compared to those without FBTCS (FBTCS−) [4, 5]. However, the underlying mechanisms and reasons for its specific prevalence among certain patient subset remain inadequately understood.

Quantitative assessments of cortical thickness and gray matter volume, as obtained through structural magnetic resonance imaging (MRI), serve as reproducible biomarkers capable of revealing in vivo morphometric changes [6]. Previous investigations have demonstrated extensive morphometric alterations in TLE patients, including pronounced atrophy within the hippocampus and temporal lobes, extending to extratemporal and subcortical regions [7, 8]. Nevertheless, rare literatures reported the morphometric alterations in TLE patients with FBTCS (FBTCS+) [9]. A recent study highlighted that brain structural network anomalies in FBTCS+ were extensive and bilateral [10] in contrast to FBTCS− where abnormalities were predominantly localized to the temporal and frontal areas.

Previous studies suggest such morphometric alterations may arise from recurrent epileptic discharges and long‐term seizures [11, 12]. However, emerging evidence challenges this perspective. For instance, longitudinal research has indicated a lack of correlation between cortical atrophy and seizure frequency [7]. Another study observed progression of gray matter atrophy also existed in seizure‐free patients [13]. Moreover, postmortem analysis has shown that recurrent seizures do not inevitably lead to neuron loss [14]. These evidences imply that the morphological changes observed in epilepsy are not fully contributed by epileptic discharges [7]. The development of brain structure is governed by a genetic context, interindividual differences in brain development are associated with several neurological disorders [15, 16]. Indeed, studies indicating similar morphometric abnormalities in asymptomatic siblings of TLE patients point toward a genetic predisposition underpinning these alterations [17]. Hence different morphometric changes between FBTCS− and FBTCS+ might reflect underlying genetic differences.

However, bridging the gap between the macroscale observations of imaging phenotypes and the microscale of gene functionality presents a considerable challenge. The Allen Human Brain Atlas (AHBA, http://human.brainmap.org) quantified more than 20,000 genes across 3702 different anatomical locations, thereby facilitating the linkage between imaging phenotypes and the transcriptome [18]. Imaging transcriptomic analysis can identify the similarity pattern between imaging phenotype and brain gene expression, which enables functional enrichment and cellular specificity analyses to elucidate the molecular mechanisms underlying these phenotypic differences [19, 20].

Our study aimed to elucidate the difference in cortical and subcortical gray matter volume (GMV) between TLE patients with FBTCS and without FBTCS. Subsequently, we employed an imaging transcriptomic analysis to identify genes associated with structural anomalies in both FBTCS+ and FBTCS−. Gene enrichment analysis was also conducted to describe the pathways and cellular organizations enriched in these related genes. This enhanced our understanding of the genetic underpinnings and molecular mechanisms contributing to the morphometric alterations in TLE patients with FBTCS.

## 2 Materials and Methods

### 2.1 Participants

This study recruited 126 unilateral TLE patients with hippocampus sclerosis (HS) who underwent presurgical evaluation at the Epilepsy Center of West China Hospital between January 2014 and February 2023. All participants underwent comprehensive diagnostic procedures including seizure semiology collection, video electroencephalography monitoring, structural MRI, and positron emission tomography (PET) if available. The inclusion criteria were: (1) age of 16 years or older; (2) diagnosis of unilateral TLE; (3) absence of abnormalities other than HS on structural MRI; (4) histopathologically confirmed HS. TLE patients were stratified into FBTCS− and FBTCS+ groups based on the occurrence of generalized tonic–clonic seizures (GTCS), with the FBTCS− group never having GTCS events in their lives and the FBTCS+ group experiencing one or more such events.

Additionally, 60 age‐ and gender‐matched healthy controls (HC) were recruited, with inclusion criteria of being 16 years or older and having no history of neurological or psychiatric disorders.

This study received ethical approval from the ethics committee of West China Hospital, and informed consent was obtained from all participants.

### 2.2 Imaging Acquisition and Preprocessing

Participants were scanned using a 3 T MRI scanner (Trio, Siemens) with an eight‐channel head coil. T1‐weighted images were acquired using 3D magnetization prepared rapid acquisition gradient echo sequence. The imaging parameters were set as follows: repetition time of 2300 ms; echo time of 4.18 ms; flip angle of 9°; field of view of 256 × 256 mm^2^; and voxel size of 1.0 × 1.0 × 1.0 mm^3^.

Data quality was ensured through visual inspection by two trained researchers. Moreover, Euler number was computed by FreeSurfer to further assess image quality quantitatively, which was propose by previous study [21]. Then to standardize the lateralization of the epileptogenic focus, T1‐weighted images of right TLE patients were flipped left to right, making the side of ictal onset in left hemisphere and labeled as ipsilateral side and right hemisphere labeled as contralateral side. Preprocessing was then conducted by using FreeSurfer pipeline [22] (version 7.3, http://surfer.nmr.mgh.harvard.edu/), which involved skull stripping, brain tissue segmentation, and construction of cortical surfaces.

### 2.3 Quantification of GMV

The cortical surface was parcellated into 308 spatially contiguous regions, with 152 in left hemisphere, based on Desikan‐Killiany (D‐K) atlas using a backtracking algorithm to minimize the influence of the parcel size [23]. Then the 308 parcellation atlas was transformed into individual space to obtain individual cortical parcellation. Subcortical segmentation was performed using the Aseg atlas from FreeSurfer. The GMV values of 322 regions (308 cortical and 14 subcortical regions) were extracted by FreeSurfer. The values of total intracranial volume (TIV) of all subjects were also extracted for further analysis.

### 2.4 Case–Control Analysis of the GMV

The linear regression model (LRM) was conducted to estimate the difference of GMV in each region between patients and HC. Firstly, the GMV values were *z*‐normalized across regions. Then, for each region of GMV (GMV_i_) the following model was used:

$${\mathrm{GMV}}_{i}=\text{intercept}+\beta_{1}\mathrm{age}\times \beta_{2}\mathrm{sex}+\beta_{3}\mathrm{TIV}+\beta_{4}\text{group}$$

The GMV_i_ was conducted as dependent variable and age, sex, and TIV as covariate variables. Finally, false discovery ratio (FDR) correction was conducted for multiple comparisons and setting *p* < 0.05 after correction to denote statistical significance.

To further contextualize the regional differences of GMV, we mapping cortical regions to Yeo network [24] and von Economo atlas [25]. We averaged the GMV values of all regions belonging to each Yeo network or von Economo atlas and then applied LRM to evaluate the difference of GMV in network level by regressing out age, sex, and TIV.

We estimated the association between regional morphometric alterations and clinical factors including seizure onset age and seizure frequency in patients. Spearman correlation was used to examine association between seizure onset age and regional GMV values for these regions with significant difference between patients and HC. Seizure frequency was categorized into two groups: frequent seizure (daily and weekly) and infrequent seizure (monthly and yearly). Then LRM was utilized to compare regional GMV values between the two seizure frequency groups, after regressing out covariates. FDR correction was conducted for multiple comparisons.

### 2.5 Preprocessing of Gene Expression Data

Gene expression profiles of postmortem brains from six healthy donors were obtained from AHBA database (http://human.brain‐map.org). We used abagen toolbox [26] (https://www.github.com/netneurolab/abagen) to process gene expression data, briefly, the processing steps including: (1) update the Montreal Neurological Institute (MNI) coordinates of AHBA tissue samples; (2) reannotation of probe to gene mapping; (3) intensity filtering with a threshold of 50% to mitigate noise; (4) choosing representative probe among those probes indexing the same gene; (5) matching AHBA tissue samples to each regions; (6) normalizing expression values of gene profile across genes and samples by a scaled robust sigmoid normalization function; (7) aggregating different samples within regions. Considering the gene expressing data of right hemisphere were obtained from only two donors, we only analyzed the left hemisphere data, which containing all six donors. Finally, the gene expression matrix of 159 regions × 15,633 genes was obtained in left hemisphere for further analysis.

### 2.6 Association of Gene Expression and Morphometric Alterations

Partial least squares (PLS) regression was applied to identify genes associated with morphometric alterations. The PLS model leveraged the *z*‐normalized gene expression matrix as the independent variable against the vector of *z*‐normalized case–control *t*‐values of GMV as the dependent variable. The first component of the PLS analysis (PLS1) was selected for its optimal representation of the case–control *t*‐values of GMV. The statistical significance of the variance explained by PLS1 was assessed through permutation testing [20]. Furthermore, to evaluate the contribution of each gene for component, we transformed the gene weight into *z*‐score value by dividing gene weight to its bootstrap standard error [20]. Then all of 15,633 genes were ranked according to *z*‐score and only genes with *p* < 0.05 after FDR correction were considered as strongly contributed genes. These genes include positive weighted genes (PLS+) and negative weighted genes (PLS−), which indicate positive or negative correlations with morphometric alterations, respectively. We explore the Spearman correlation between case–control *t*‐values and highest rank genes of PLS+ and PLS−, *p* value was estimated by spin test, which is a spatial permutation method to correct effects of spatial autocorrelation [27].

To assess the sensitivity of PLS genes, we compared PLS genes and a gene list of TLE reported by Guelf et al. [28], which was identified by whole transcriptome on temporal cortical specimens from TLE with HS patients who underwent surgical resection. This gene list including 3016 highly expressed (i.e., upregulated) genes and 3087 lowly expressed (i.e., downregulated) genes with *p* < 0.05 after FDR correction. We calculated the enrichment ratio (ER) to quantify whether these dysregulated genes were enriched in PLS+ or PLS− genes. Specifically, the ER was calculated by difference of median weight of intersection genes between PLS+ genes and TLE gene list and the mean median weight of the same number of intersection genes after 10,000 randomly permutation, which was then divided by the standard deviation of the permuted genes. Significance was determined by the proportion of median weight of intersection genes relative to 10,000 randomly permuted intersection genes. The statistical significance was set at *p* < 0.05. This approach also extended to examining the specificity of PLS genes across a spectrum of brain disorders including major depressive disorder (MDD), autism spectrum disorder (ASD), schizophrenia (SCZ), bipolar disorder (BD), and alcohol abuse disorder (AAD), which identified in research by Gandal et al. [29].

### 2.7 Pathway Enrichment Analysis

We applied Metascape (http://metascape.org/), a web‐based gene set analysis toolkit, to conduct Gene Ontology (GO) biological process pathway and Kyoto Encyclopedia of Genes and Genomes (KEGG) in PLS+ genes. To identify the shared or unique enrichment pathways, a multiple‐gene list analysis was performed containing both significant genes of FBTCS− and FBTCS+.

### 2.8 Cell Types Assignment

To explain the difference of FBTCS− and FBTCS+ in cellular perspective, we assigned PLS+ genes to each brain cell type. To get gene sets of brain cell types, data from five different single cell studies [30, 31, 32, 33, 34] were compiled to avoid bias based on acquisition methodology, analysis, or thresholding. Then following the procedure of Seidlitz et al. [35], the initial inclusion of 58 cell classes were subsequently classified into seven canonical classes, comprising astrocytes, endothelial cells, excitatory neurons, inhibitory neurons, microglia, oligodendrocytes, and oligodendrocyte precursor cells (OPCs). We also calculated ER to assess which cell type was significantly enriched in the PLS genes.

### 2.9 Relationship Between GABA Neurotransmitter and GMV

The high‐resolution atlas of GABAergic distribution was generated by Nørgaard et al. [36], which based on in vivo [11C] flumazenil PET data from healthy participants. Following the methodology of Hasson et al. [37] we parcellated the GABAergic atlas into 322 parcels and extracted the value of GABAergic for each region. Spearman correlations between the case–control *t*‐values of GMV and GABA energetic values were conducted. *p* < 0.05 after spin test was considered as statistical significance.

### 2.10 Subgroup Analysis

Previous study had demonstrated different history of FBTCS displayed different network connection mode [38]. Hence, we divided the FBTCS+ group into two subgroups: Patients with a history of FBTCS but no episodes in over 2 years were categorized into the remote‐FBTCS+ subgroup, while those with recurrent FBTCS episodes within the past 2 years were categorized as the current‐FBTCS+ subgroup.

### 2.11 Statistical Analysis

Baseline demographic and clinical data were analyzed using IBM SPSS (version 25). Categorical variables were compared using the chi‐square test. For continuous variables, normality was first assessed using the Shapiro–Wilk test. If the data were normally distributed, the Student *t*‐test was used; otherwise, differences between groups were assessed using the Mann–Whitney *U* test or the Kruskal–Wallis *H* test. A two‐tailed *p* < 0.05 was considered as statistically significant.

## 3 Results

### 3.1 Baseline Demographic and Clinical Data

No significance was found on Euler number comparison among FBTCS−, FBTCS+ and HC (*F* = 1.187, *p* = 0.307, Figure S1). The baseline demographic and clinical data in this study are shown in Table 1.

**TABLE 1:** Demographic and clinical characteristics.

Variables	FBTCS− (*n* = 37)	FBTCS+ (*n* = 89)	HC (*n* = 60)	*p*
Age, median [IQR], years	25 [21, 28]	25 [22, 31]	24 [20, 29]	0.209*
Gender, *n* (%)				0.243^#^
Male	17 (45.9)	51 (57.3)	38 (63.3)	
Female	20 (54.1)	38 (42.7)	22 (36.7)	
TIV, median [IQR], cm^3^	1465.20 [1360.96, 1556.85]	1480.50 [1411.39, 1602.28]	1534.40 [1445.34, 1644.40]	0.075*
Lateralization, *n* (%)				0.936^#^
Left	19 (51.4)	45 (50.6)	NA	
Right	18 (48.6)	44 (49.4)	NA	
Febrile seizure history, *n* (%)				0.051^#^
Yes	15 (40.5)	53 (59.6)	NA	
No	22 (59.5)	36 (40.4)	NA	
Seizure frequency, *n* (%)				0.704^#^
Frequent	19 (27.9)	49 (72.1)	NA	
Infrequent	18 (31.0)	40 (69.0)	NA	
Seizure onset age, median [IQR], years	15 [9, 18]	14 [8, 18]	NA	0.332^§^
Duration, median [IQR], years	10 [6, 14]	12 [7, 17]	NA	0.155^§^

Abbreviations: FBTCS−, without focal to bilateral tonic–clonic seizures; FBTCS+, with focal to bilateral tonic–clonic seizures; HC, healthy control; IQR, interquartile range; TIV, total intracranial volume.

* Kruskal–Wallis *H* test.

^#^ Chi‐square test.

^§^ Mann–Whitney *U* test.

### 3.2 Case–Control Differences of Regional GMV

**FIGURE 1:**
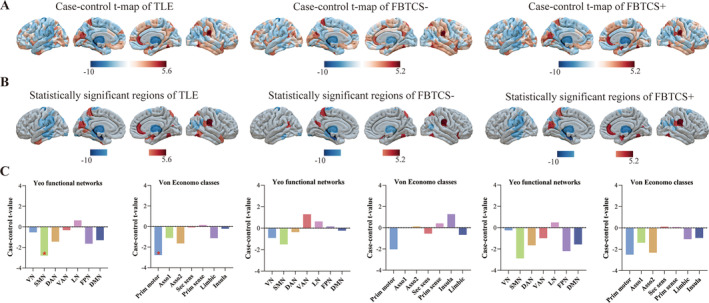
Differences in GMV. (A) Case–control t‐map of regional GMV in TLE (left), FBTCS− (middle) and FBTCS+ (right) versus HC. (B) Statistical significant regions in TLE (left), FBTCS− (middle), and FBTCS+ (right) versus HC after FDR correction. (C) The differences in Yeo functional networks and von Economo classes in TLE (left), FBTCS− (middle) and FBTCS+ (right). Asso1, association cortex1; Asso2, association cortex 2; DAN, dorsal attention network; DMN, default mode network; FPN, frontoparietal network; FBTCS−, without focal to bilateral tonic–clonic seizures; FBTCS+, with focal to bilateral tonic–clonic seizures; GMV, gray matter volume; LN, limbic network; Prim motor, primary motor cortex; Prim sens, primary sensory cortex; Sec sens, second sensory cortex; SMN, somato‐motor network; TLE, temporal lobe epilepsy; VAN, ventral attention network; VN, visual network.

The differences in regional GMV values were estimated by applying LRM after regressing out age, sex, and TIV in Figure 1A. The atrophy regions in TLE compared to HC were mainly in ipsilateral temporal lobe, parahippocampal gyrus, precentral gyrus, postcentral gyrus, precuneus, superior parietal gyrus, supramarginal gyrus, insula, thalamus, striatum, and hippocampus and contralateral inferior parietal gyrus, superior parietal gyrus, thalamus, and putamen (all *p*_FDR_ < 0.05, Figure 1B left and Table S1). Post hoc analysis showed that the regional GMV alterations between FBTCS+ and HC were similar with TLE, mainly involved in ipsilateral temporal lobe, parietal lobe, subcortical regions, as well as contralateral cortical and subcortical regions (all *p*_FDR_ < 0.017, Figure 1B right and Table S2). While FBTCS− patients displayed milder alteration in regional GMV, the atrophy regions mainly included temporal lobe, parahippocampal gyrus, precentral gyrus, lateral occipital gyrus, subcortical regions, and contralateral inferior parietal gyrus (all *p*_FDR_ < 0.017, Figure 1B middle and Table S3). No difference was found between FBTCS+ and FBTCS− groups.

Two prior classification of cortical regions, Yeo network and von Economo atlas, were mapped to 322 regions (Figure 1C). We only found significant atrophy in SMN of Yeo network and primary motor cortex of von Economo atlas among TLE when compared to HC (both *p*_FDR_ < 0.05, Figure S2 and Tables S4 and S5). No significant difference was found after post hoc analysis (Tables S6–S9).

We estimated relationship between clinical factors and significant regional GMV in FBTCS− and FBTCS+. We only found superior temporal gyrus was positively correlated with seizure onset age in FBTCS+ (*r* = 0.378, *p*_FDR_ = 0.011, Table S11).

### 3.3 Brain Gene Expression Related to Altered GMV

**FIGURE 2:**
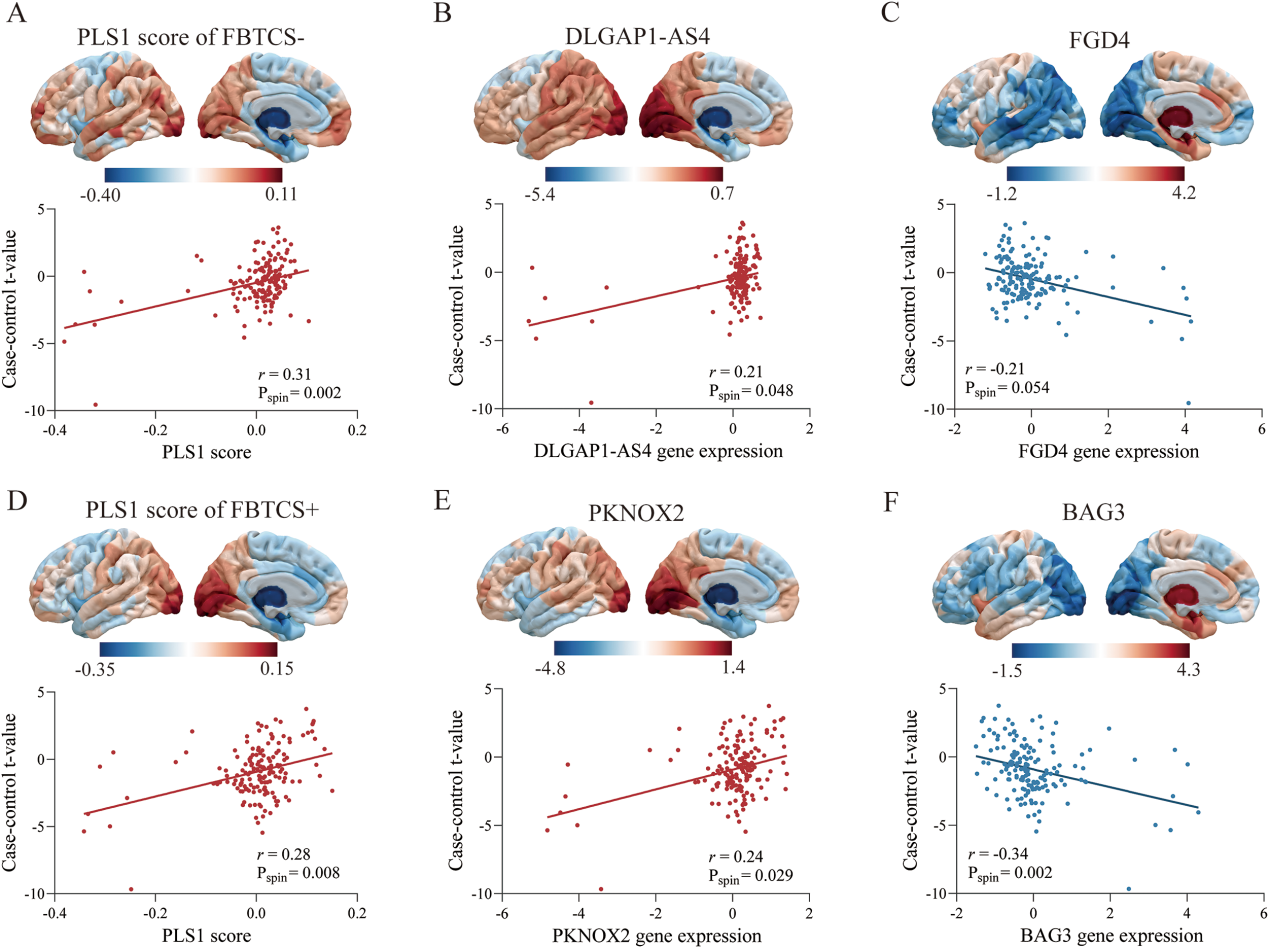
Association between regional gene expression profiles and case–control differences of gray matter volume. (A) The scatter plot shows the regional PLS1 scores was correlated with case–control *t*‐values in left hemisphere of FBTCS−, with each dot representing a brain region. (B) The regional expression of the most positively weighted gene in left hemisphere and the correlation with case–control *t*‐value of FBTCS−. (C) The most negatively weighted gene and the negative correlation with case–control *t*‐value of FBTCS−. (D) The regional PLS1 scores and was positively correlated with case–control *t*‐value in left hemisphere of FBTCS+. (E) The regional expression of the most positively weighted gene in left hemisphere and was positive correlated with case–control *t*‐value of FBTCS+. (F) The regional expression of the most negatively weighted gene and was negatively correlated with case–control *t*‐value of FBTCS+. FBTCS−, without focal to bilateral tonic–clonic seizures; FBTCS+, with focal to bilateral tonic–clonic seizures; PLS1, the first component of the PLS analysis.

The PLS1 explained 17.1% variance of GMV alterations in FBTCS− (*p*_perm_ = 0.001) and 14.0% in FBTCS+ (*p*_perm_ = 0.007). The PLS1 score map was positively correlated with the case–control t‐map of FBTCS− (*r* = 0.31, *p*_spin_ = 0.002, Figure 2A) and FBTCS+ (*r* = 0.28, *p*_spin_ = 0.008 Figure 2D), respectively.

By ranking the weights of PLS1 genes according to z‐score, we found that FBTCS− was related to 2155 PLS+ genes and 2012 PLS− genes, and FBTCS+ was related to 2167 PLS+ genes and 1818 PLS− genes (all *p*_FDR_ < 0.05). The top‐ranked genes, *DLGAP1‐AS4* in PLS+ and *FGD4* in PLS− genes, were correlated with the case–control t‐map in the FBTCS− group (*r* = 0.21, *p*_spin_ = 0.048; *r* = −0.21, *p*_spin_ = 0.054, respectively, Figure 2B,C). Similarly, the corresponding genes *PKNOX2* and *BAG3* were significantly correlated with morphometric alterations in FBTCS+ group (*r* = 0.24, *p*_spin_ = 0.029; *r* = −0.34, *p*_spin_ = 0.002, respectively, Figure 2E,F).

Gene enrichment analysis found that these downregulated genes from TLE gene list were both significantly enriched in PLS+ genes of FBTCS− (ER = 6.76, *p*_FDR_ = 0.001) and FBTCS+ (ER = 7.69, *p*_FDR_ = 0.001, Table S12). Furthermore, ASD downregulated genes were also enriched in PLS+ genes of FBTCS− (ER = 2.70, *p*_FDR_ = 0.007) and FBTCS+ (ER = 2.97, *p*_FDR_ = 0.019, Table S12). The PLS− genes of FBTCS+ were enriched in the upregulated TLE gene list (ER = 3.97, *p*_FDR_ = 0.001), while the PLS− genes of FBTCS− showed no enrichment in the TLE‐related gene list, suggesting lower sensitivity for PLS− genes. Furthermore, the PLS− genes of FBTCS+ were also enriched in other brain disorders, including ASD (ER = 2.62, *p*_FDR_ = 0.018), SCZ (ER = 2.02, *p*_FDR_ = 0.049), and MDD (ER = 2.27, *p*_FDR_ = 0.049), indicating lower specificity when compared to PLS+ genes (Table S13). Based on the above findings, we focused on PLS+ genes for subsequent analysis.

### 3.4 Pathway Enrichment of PLS+ Genes

**FIGURE 3:**
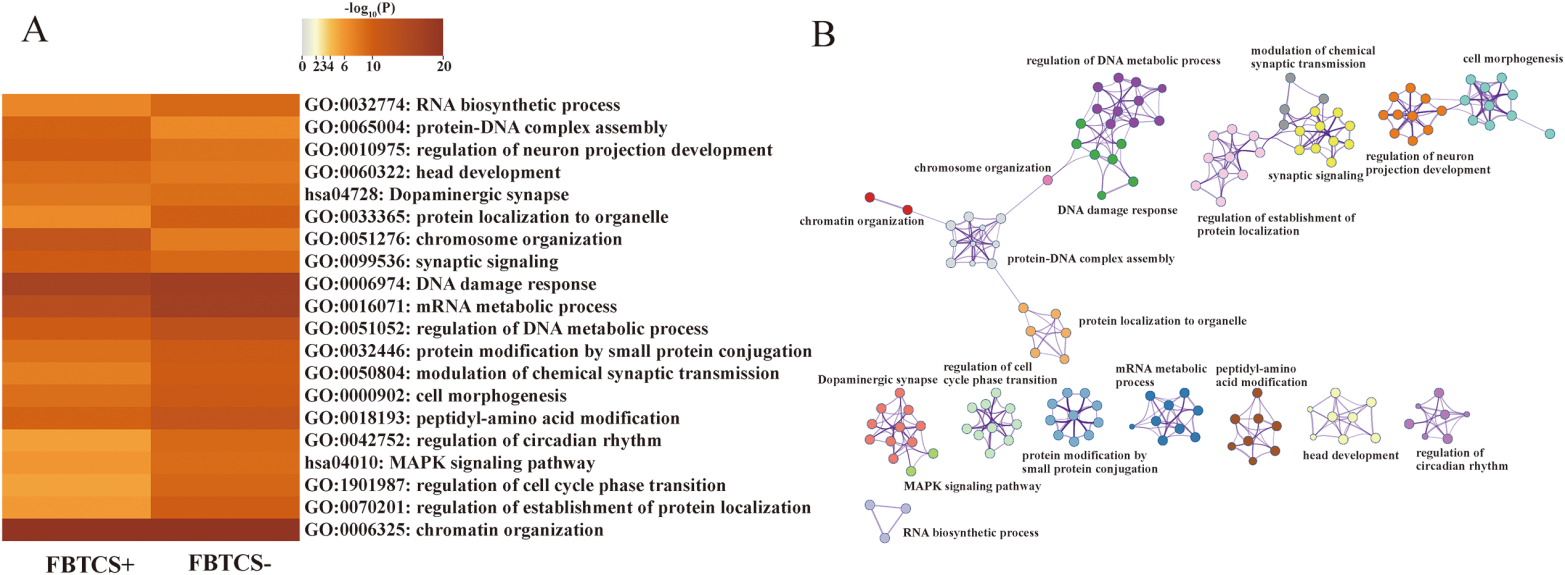
Pathway Enrichment analysis of the PLS+ genes. (A) The heatmap displayed the GO and KEGG pathways for PLS+ genes. (B) Network of enriched GO and KEGG terms by metascape. GO, gene ontology; KEGG; Kyoto Encylopedia of Gene, Genomes.

The web‐based gene set analysis toolkit, Metascape, was used for functional annotations, including GO biological processes and KEGG pathways. To identify the shared or unique functional annotations, we combined PLS+ genes of FBTCS− and FBTCS+ to perform multiple‐gene list analysis. We found that shared enrichment terms of biological processes and KEGG pathway mainly centered around neural development and synaptic function, such as “regulation of neuron projection development,” “synaptic signaling,” “dopaminergic synapse,” and “modulation of chemical synaptic transmission,” it also enriched in epigenetic regulation, including “chromosome organization,” “chromatin organization,” “DNA damage response,” moreover, mitogen‐activated protein kinases (MAPK) signaling pathway was also found as shared KEGG pathway term (Figure 3). Although most of the terms overlapped between the FBTCS− and FBTCS+, there were also exist differences. For instance, FBTCS− had unique GO terms mainly in catabolic processes, such as “autophagy,” “regulation of cellular catabolic process.” While the unique terms in FBTCS+ was “regulation of smoothened signaling pathway” (File S1).

### 3.5 Cell Type Signature of PLS+ Genes

**FIGURE 4:**
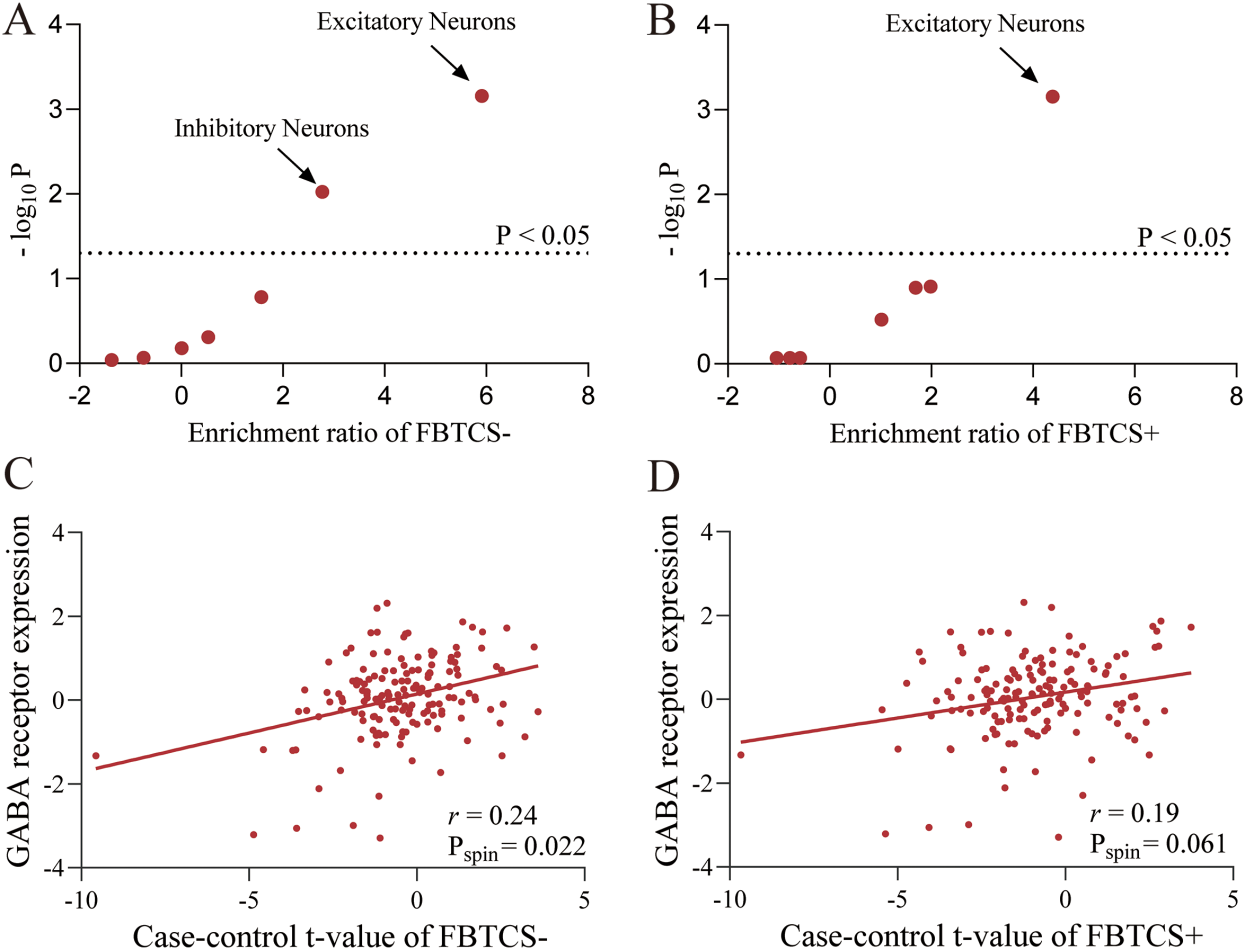
Cell type enrichment of PLS+ genes and correlation between regional GABA receptor expression and case–control *t*‐value. (A) Excitatory and inhibitory neurons were significantly enriched in FBTCS− patients. (B) Only excitatory neurons were significantly enriched in in FBTCS+ patients. (C) The scatter plot shows the regional GABA receptor expression was significantly correlated with case–control *t*‐value of FBTCS−, with each dot representing a brain region. (D) No significant correlation was found between GABA receptor expression and case–control *t*‐value of FBTCS+. FBTCS−, without focal to bilateral tonic–clonic seizures; FBTCS+, with focal to bilateral tonic–clonic seizures.

To further explore the GMV changes related genes in cellular perspective, we assigned PLS+ genes to seven canonical cell types based on previous studies [30, 31, 32, 33, 34, 35]. We found PLS+ genes in FBTCS− enriched in excitatory neurons (ER = 5.91, *p*_perm_ = 0.001) and inhibitory neurons (ER = 2.77, *p*_perm_ = 0.009, Figure 4A), while FBTCS+ group only enriched in excitatory neurons (ER = 4.39, *p*_perm_ = 0.001, Figure 4B, Table S14). Considering most inhibitory neurons communicate by the neurotransmitter of GABA, we applied a brain GABA energetic atlas based on in vivo [11C] flumazenil PET reported by Nørgaard et al. [36] and found the case–control t‐map of FBTCS− was positively correlated with GABA receptor expression (*r* = 0.24, *p*_spin_ = 0.022, Figure 4C). However, no significant correlation was found between case–control t‐map and GABA receptor expression in FBTCS+ (Figure 4D).

### 3.6 Subgroup Analysis

We also applied LRM to estimate differences in regional GMV values between current‐FBTCS+, remote‐FBTCS+, and HC (Figure S3A). The distribution of morphometric alterations in current‐FBTCS+ was similar to that observed in FBTCS+ group, with significant atrophy in the ipsilateral temporal lobe, supramarginal gyrus, and subcortical regions (Figure S3B left, Table S15). In contrast, the atrophy regions in remote‐FBTCS+ were more localized, affecting the ipsilateral superior temporal lobe, parahippocampal gyrus, precentral gyrus, superior parietal gyrus, supramarginal gyrus, hippocampus, and putamen (Figure S3B right, Table S16).

We compared the PLS+ genes of current‐FBTCS+ and remote‐FBTCS+ patients with those of FBTCS− patients and found a 77.9% overlap between FBTCS− and remote‐FBTCS+ genes, compared with a 53.4% overlap between FBTCS− and current‐FBTCS+ genes (Figure S4). We created a multiple‐gene list containing PLS+ genes from FBTCS−, remote‐FBTCS+, and current‐FBTCS+ and applied enrichment analysis. The shared enrichment terms were similar with abovementioned results of FBTCS− and FBTCS+ (Figure S5). Interestingly, remote‐FBTCS+ shared unique GO terms related to catabolic processes with FBTCS−, which were not found in current‐FBTCS+ (File S2).

We then assigned PLS+ genes of remote‐FBTCS+ and current‐FBTCS+ to seven canonical cell types, PLS+ genes in remote‐FBTCS+ were enriched in excitatory neurons (ER = 6.02, *p*_perm_ = 0.001) and inhibitory neurons (ER = 2.66, *p*_perm_ = 0.011, Figure S6), similar to the FBTCS− group. In contrast, the current‐FBTCS+ group showed enrichment only in excitatory neurons (ER = 3.79, *p*_perm_ = 0.001, Figure S6, Table S17). No significant correlation was found between case–control t‐map and GABA receptor expression in either remote‐FBTCS+ or current‐FBTCS+ groups.

## 4 Discussion

In the present study, we investigated the abnormalities of morphometry in TLE patients with FBTCS− and FBTCS+. Our findings indicated significant morphometric alterations in both bilateral neocortical and subcortical regions in FBTCS+, whereas those with FBTCS− exhibited more localized changes. Furthermore, imaging transcriptomic analysis recognized morphometric alterations associated genes and further found PLS+ genes associated with neurobiological relevant pathways and different cellular expression preferences between FBTCS− and FBTCS+. Moreover, subgroup analysis revealed heterogeneity within the FBTCS+ group. The current‐FBTCS+ subgroup displayed morphometric alterations and cellular signatures similar to those found in the overall FBTCS+ group, while the remote‐FBTCS+ subgroup exhibited features more akin to the FBTCS− group. These findings revealed different brain morphological profiles and uncovered genetic variances underlying FBTCS− and FBTCS+.

Morphometric abnormalities in TLE patients had been well demonstrated in extensive neuroimaging studies, with the alteration pattern covering from medial temporal lobe to extensive extratemporal regions [7, 8, 12]. A recent longitudinal study observed widespread and progressive cortical atrophy in TLE patients with the most pronounce atrophy in ipsilateral hemisphere of the epileptic focus but also extending to the contralateral hemisphere [7], which were in line with our findings. We further found widespread and bilateral morphometric abnormalities in FBTCS+, in contrast more localized ipsilateral cortical atrophy in FBTCS−, which was similar to previous study [10]. These evidences suggested FBTCS+ had more severe neuronal damages, causing bilateral cortical atrophy. Notably, atrophy of subcortical regions such as thalamus, stratum, and pallidum were also found, indicating the important role of thalamocortical and basal ganglia‐thalamocortical loops in the pathological network of TLE [38].

Despite the brain morphology alteration of TLE has been extensively studied, building association between macroscale neuroimaging findings and genetic architecture of epilepsy to investigate molecular mechanism of macroscale alteration is rarely explored. By applying imaging transcriptomic analysis, we found that morphometric alterations were spatially correlated with AHBA gene expression and identified PLS+ genes that could maximum explained morphometric alterations in FBTCS− and FBTCS+. We found PLS+ genes of both FBTCS− and FBTCS+ marked enriched for downregulated genes from a whole transcriptomic study [28], which further validate sensitivity of imaging‐related genes. Specificity of these associations was supported by validating PLS+ genes with other brain disorders and only found downregulated genes of ASD were enriched. It is well known that comorbid psychiatric symptoms are common in TLE patients. Previous studies have reported a high prevalence of ASD (8.1%) among epilepsy patients, which is seven times higher than people without epilepsy [39] and found shared mechanisms such as synaptic plasticity, neuronal morphology, proliferation, and migration between epilepsy and ASD [40]. Hence, shared genetic risk between TLE and ASD may explain high psychotic comorbidity and shared molecular mechanisms.

By applying pathway enrichment analysis, we found FBTCS− and FBTCS+ had shared GO and KEGG terms in synaptic function, epigenetic regulation, and MAPK signaling pathway. Synapses are essential components of neurons, which play a key role in transmission of nervous impulses from one neuron to another. Pre‐ and postsynapses is mainly mediated by glutamate in excitatory synapses and by GABA for inhibitory synapses, respectively [41]. Potential excitation–inhibition imbalance in the generation of seizures is commonly accepted as one of the major causes of epilepsy [42]. Epigenetic regulation refers to the control of gene expression through changes in the structure of chromatin and was considered as a key feature of neurodevelopment, brain maturation, and adult brain function [43]. Recently, several evidence had elucidated epigenetic mechanisms to be involved in epileptogenesis and progression of the TLE and may represent potential biomarkers [1, 43]. The function of MAPK signaling pathway is to transduce signals from a receptor on the surface of the cell to the cell nucleus and activated for cell growth, division, and differentiation [44]. Recently a study used whole‐exome and gene‐panel sequencing on hippocampus from drug‐resistant TLE patients and identified 11 pathogenic somatic variants in hippocampus, most of these variants constitutively activate MAPK signaling [45]. Salman et al. [46] also found differential expression of the MAPK signaling pathway by applying microarray on hippocampus of TLE patients. These studies suggested that MAPK signaling pathway may contribute to the pathogenesis of TLE and highlighted new therapeutic targets in the future. Apart from shared pathway terms, we also found unique terms in FBTCS− and FBTCS+, the former including catabolic processes and the latter was regulation of smoothened signaling pathway. Autophagy is an explicit cellular process for degradation and recycling of misfolding and damaging organelles and participating in synaptic homeostasis and regulation of neurotransmitters in brain [47]. Dysfunctional autophagic process was closely related to development and progression of epilepsy, for example, it had been observed defective autophagy and excessive activated the mammalian target of rapamycin (mTOR) signaling, an important negative regulator of autophagy, in focal cortical dysplasia, tuberous sclerosis complex and in TLE [48, 49, 50]. Animal experiments had observed inhabitation of autophagy could disturbing excitatory/inhibitory (E/I) balance by inducing neuronal hyper‐excitability and reducing of GABAergic inhibitory current in the hippocampus [51]. In our study, we found FBTCS‐related genes enriched on autophagy while it was not in FBTCS+, it may reflect loss of autophagy function may disturb the E/I balance and promote generalization of epileptic discharges.

The decrease in GMV observed on MRI reflects the loss of neurons at the microscale level. Previous animal had observed GABAergic neuronal loss in neocortex and hippocampus [52, 53]. Ictal events in the TLE are commonly manifested by an imbalance of chemical excitatory and inhibitory signaling. The loss of GABAergic neurons may shift the balance of excitation and inhibition toward excitation, which had been considered as a critical cause of seizure activity in epileptic models of TLE [54]. Accordingly, some AEDs by directly allosterically binding with GABA receptors, or enhancing GABA receptor‐mediated chloride ion currents, or by modifying GABA transporters and the activity of enzymes to enhance inhibitory neurotransmission [55]. Some latest experimental therapeutic strategies attempted new approach to enhance inhibition to regulate epileptic discharges. For instance, Krook‐Magnuson et al. [56] by using an optogenetic method to activate a subtype of GABAergic neurons and found it can stop seizures rapidly. Moreover, researchers have found transplanting inhibitory neuron precursor into neonatal or adult neocortex or hippocampus can ameliorate electrophysiological and behavioral abnormalities [57]. The above studies indicate that activation of GABAergic neurons plays an important role in inhibiting the onset of epilepsy, and there are also studies indicating that it plays a key role in the generalization of discharges from focal onset of TLE. An optogenetic study observed photoactivation GABAergic neurons in subicular could retarded FBTCS+ by inhibiting the firing of pyramidal neurons in mouse models [58]. Consistent with previous studies, by applying imaging transcriptome we found distinct cellular characterization in FBTCS− and FBTCS+. The significant enrichment of FBTCS− related genes was most pronounced in excitatory and inhibitory neuron, while FBTCS+ related genes only enriched in excitatory neuron. No significant correlation between FBTCS+ and GABA energetic atlas further confirm this result. It indicated that attenuate GABAergic neuron may contribute to the generalization of epileptic discharges.

Several limitations needed considered in present study. Firstly, AHBA transcriptome data were measured from postmortem in six adult brains but not patients with TLE. Currently, we lack transcriptional data from TLE patients who have undergone surgery. To validate the sensitivity of PLS genes, we compared them with a gene list from a whole transcriptome analysis of TLE patients who underwent surgical resection reported by Guelf et al. [28]. We found that downregulated genes from the TLE gene list were significantly enriched in the PLS+ genes of both FBTCS− and FBTCS+ patients, further proving the reliability of our results. Secondly, the GABAergic atlas used in this study is derived from healthy participants. Future research should include PET imaging of GABAergic receptors in TLE patients to uncover potential differences in the distribution of inhibitory neurotransmitters between FBTCS+ and FBTCS−. Thirdly, consistent with previous studies, considering the microarray expression data of right hemisphere in AHBA dataset only captured from two adult brains, we only selected data from left hemisphere [20, 59]. We flipped the right hemisphere to left in right TLE patients, hence the genes associated with imaging phenotype only indicated ipsilateral hemisphere morphometric alterations. Moreover, to avoid the influence of confounding factors, we adjusted for gender, age, and TIV in the linear regression model. However, it might not fully address and some potential confounders we not considered, which might influence our findings. Finally, as previous studies mentioned, atrophy of cortical and subcortical regions in TLE were progressive, this cannot be identified in our cross‐sectional data and unable to identify the specific genes that play a role in progressive atrophy. Therefore, a further imaging transcriptomic analysis with longitudinal data is needed.

## 5 Conclusion

In summary, this study explores the link between morphometric alterations and gene expression in TLE patients with and without FBTCS. Enrichment analysis identified both shared and distinct neurobiological pathways in FBTCS+ and FBTCS−. Abnormalities in inhibitory neurons and a weak association between GABAergic receptors and morphometric changes in FBTCS+ suggest that GABA inhibition deficiency may underlie the increased susceptibility of TLE patients to FBTCS. Overall, our findings offer new insights into the genetic and molecular mechanisms driving morphometric alterations in FBTCS.

**Abbreviations**
AAD: alcohol abuse disorder
AHBA: Allen Human Brain Atlas
ASD: autism spectrum disorder
BD: bipolar disorder
D‐K atlas: Desikan‐Killiany atlas
E/I: excitatory/inhibitory
FBTCS+: with focal to bilateral tonic–clonic seizures
FBTCS−: without focal to bilateral tonic–clonic seizures
FDR: false discovery ratio
GMV: gray matter volume
GO: Gene Ontology
GTCS: generalized tonic–clonic seizures
HC: healthy controls
HS: hippocampus sclerosis
KEGG: Kyoto Encyclopedia of Genes and Genomes
LRM: linear regression model
MAPK: mitogen‐activated protein kinases
MDD: major depressive disorder
MRI: magnetic resonance imaging
MNI: Montreal Neurological Institute
mTOR: the mammalian target of rapamycin
OPCs: oligodendrocyte precursor cells
PET: positron emission tomography
PLS: partial least squares
PLS1: first component of the PLS
PLS+: positive weighted genes of PLS
PLS−: negative weighted genes of PLS
SCZ: schizophrenia
TIV: total intracranial volume
TLE: temporal lobe epilepsy

## Author Contributions

Q.L. and D.A. formulated the study design. Q.L. and D.A. performed the literature search, data analysis, data interpretation, and wrote the paper. Q.L. performed data analysis. W.L., Y.L., P.L., Y.Z., X.H., K.H., and D.C. helped the collection of clinical and neuroimaging data. Q.G. and D.Z. consulted on data analysis and helped data interpretation. Q.L. and D.A. verified the underlying data.

## Ethics Statement

We confirm that we have read the Journal's position on issues involved in ethical publication and affirm that this report is consistent with those guidelines.

## Conflicts of Interest

The authors declare no conflicts of interest.

## Supporting information


File S1.

File S2.

Data S1.

## Data Availability

The data that support the findings of this study are available from the corresponding author upon reasonable request. The codes for PLS analysis are openly available at https://github.com/SarahMorgan/Morphometric_Similarity_SZ. The code for spatial permutation testing can be found at https://github.com/frantisekvasa/rotate_parcellation. The Metascape tool of gene enrichment analysis was available at http://metascape.org/. Statistical analyses were conducted using MATLAB (version: R2016b) and SPSS Statistics (version: 25.0).
