# Supplementary material for: Brain Morphometric Alterations in Focal to Bilateral Tonic–Clonic Seizures in Epilepsy Associated With Excitatory/Inhibitory Imbalance
Source: CNS Neurosci Ther. 2024 Nov 24;30(11):e70129. doi: 10.1111/cns.70129 (PMC11586465; doi:10.1111/cns.70129)
Supplement: Supplementary file 3 — Data S1. [file CNS-30-e70129-s001.docx]

Supplementary Table 1: Statistically significant regional difference between TLE and HC.

| Regions | t-statistic^a^ | P_FDR_^b^ |
| --- | --- | --- |
| lh_cuneus_part1 | 3.71 | 2.78×10^-4^ |
| lh_inferiortemporal_part1 | 2.80 | 5.65×10^-3^ |
| lh_inferiortemporal_part6 | -3.99 | 9.46×10^-5^ |
| lh_lateraloccipital_part8 | -3.20 | 1.63×10^-3^ |
| lh_lingual_part5 | 2.77 | 6.28×10^-3^ |
| lh_middletemporal_part4 | -3.31 | 1.14×10^-3^ |
| lh_middletemporal_part5 | -4.33 | 2.48×10^-5^ |
| lh_parahippocampal_part2 | -4.01 | 9.04×10^-5^ |
| lh_pericalcarine_part2 | 2.74 | 6.73×10^-3^ |
| lh_postcentral_part4 | -3.07 | 2.49×10^-3^ |
| lh_precentral_part3 | -4.28 | 2.99×10^-5^ |
| lh_precuneus_part1 | -2.85 | 4.82×10^-3^ |
| lh_precuneus_part2 | 3.34 | 1.03×10^-3^ |
| lh_precuneus_part3 | 3.39 | 8.50×10^-4^ |
| lh_precuneus_part7 | -3.11 | 2.19×10^-3^ |
| lh_superiorparietal_part3 | -2.76 | 6.33×10^-3^ |
| lh_superiorparietal_part6 | -3.03 | 2.84×10^-3^ |
| lh_superiortemporal_part1 | -5.29 | 3.57×10^-7^ |
| lh_superiortemporal_part3 | -3.77 | 2.18×10^-4^ |
| lh_supramarginal_part2 | -3.15 | 1.93×10^-3^ |
| lh_supramarginal_part3 | -4.05 | 7.47×10^-5^ |
| lh_supramarginal_part4 | -3.05 | 2.63×10^-3^ |
| lh_supramarginal_part5 | -4.13 | 5.55×10^-5^ |
| lh_supramarginal_part6 | -3.83 | 1.76×10^-4^ |
| lh_insula_part3 | -4.15 | 5.10×10^-5^ |
| lh_thalamus | -5.82 | 2.70×10^-8^ |
| lh_caudate | -2.93 | 3.82×10^-3^ |
| lh_putamen | -5.06 | 1.01×10^-6^ |
| lh_pallidum | -4.66 | 6.18×10^-6^ |
| lh_hippocampus | -10.21 | 1.37×10^-19^ |
| rh_fusiform_part3 | 4.63 | 6.90×10^-6^ |
| rh_inferiorparietal_part3 | -3.71 | 2.73×10^-4^ |
| rh_inferiorparietal_part5 | -3.10 | 2.23×10^-3^ |
| rh_inferiorparietal_part7 | -3.16 | 1.83×10^-3^ |
| rh_inferiortemporal_part2 | 2.98 | 3.32×10^-3^ |
| rh_rostralanteriorcingulate_part1 | 3.92 | 1.28×10^-4^ |
| rh_superiorfrontal_part5 | -3.07 | 2.50×10^-3^ |
| rh_superiorparietal_part1 | -3.12 | 2.13×10^-3^ |
| rh_superiorparietal_part7 | 3.09 | 2.32×10^-3^ |
| rh_superiortemporal_part1 | 2.89 | 4.39×10^-3^ |
| rh_supramarginal_part2 | 3.87 | 1.51×10^-4^ |
| rh_supramarginal_part4 | 5.56 | 9.69×10^-8^ |
| rh_supramarginal_part5 | 3.15 | 1.92×10^-3^ |
| rh_thalamus | -5.50 | 1.26×10^-7^ |
| rh_putamen | -3.04 | 2.73×10^-3^ |
| rh_amygdala | 3.41 | 7.98×10^-4^ |

^a^t-statistics were calculated by general linear model after regressing out age, sex, and TIV.

^b^All P values were corrected by BH-FDR.

lh, left hemisphere; rh, right hemisphere; BH-FDR, Benjamini-Hochberg false discovery rate; TLE, temporal lobe epilepsy.

Supplementary Table 2: Statistically significant regional difference between FBTCS+ and HC.

| Regions | t-statistic^a^ | P_FDR_^b^ |
| --- | --- | --- |
| lh_cuneus_part1 | 3.74 | 2.61×10^-4^ |
| lh_inferiortemporal_part6 | -3.38 | 9.23×10^-4^ |
| lh_middletemporal_part4 | -3.40 | 8.80×10^-4^ |
| lh_middletemporal_part5 | -4.00 | 1.02×10^-4^ |
| lh_parahippocampal_part2 | -3.40 | 8.61×10^-4^ |
| lh_pericalcarine_part2 | 2.85 | 5.07×10^-3^ |
| lh_postcentral_part4 | -3.11 | 2.23×10^-3^ |
| lh_precentral_part3 | -3.43 | 7.88×10^-4^ |
| lh_precuneus_part1 | -3.05 | 2.69×10^-3^ |
| lh_precuneus_part2 | 2.96 | 3.62×10^-3^ |
| lh_precuneus_part3 | 2.78 | 6.13×10^-3^ |
| lh_precuneus_part7 | -3.12 | 2.20×10^-3^ |
| lh_superiorparietal_part3 | -2.84 | 5.23×10^-3^ |
| lh_superiorparietal_part6 | -2.81 | 5.72×10^-3^ |
| lh_superiortemporal_part1 | -5.46 | 2.07×10^-7^ |
| lh_superiortemporal_part3 | -3.84 | 1.87×10^-4^ |
| lh_supramarginal_part2 | -3.42 | 8.04×10^-4^ |
| lh_supramarginal_part3 | -4.73 | 5.43×10^-6^ |
| lh_supramarginal_part4 | -3.19 | 1.73×10^-3^ |
| lh_supramarginal_part5 | -4.25 | 3.80×10^-5^ |
| lh_supramarginal_part6 | -3.51 | 6.01×10^-4^ |
| lh_insula_part3 | -4.35 | 2.52×10^-5^ |
| lh_Thalamus | -5.36 | 3.28×10^-7^ |
| lh_Caudate | -2.89 | 4.51×10^-3^ |
| lh_Putamen | -4.99 | 1.73×10^-6^ |
| lh_Pallidum | -4.06 | 8.00×10^-5^ |
| lh_Hippocampus | -9.67 | 2.45×10^-17^ |
| rh_bankssts_part2 | -2.98 | 3.43×10^-3^ |
| rh_fusiform_part3 | 4.29 | 3.32×10^-5^ |
| rh_inferiorparietal_part3 | -3.81 | 2.02×10^-4^ |
| rh_inferiorparietal_part7 | -3.06 | 2.67×10^-3^ |
| rh_rostralanteriorcingulate_part1 | 3.87 | 1.65×10^-4^ |
| rh_superiorfrontal_part5 | -3.23 | 1.55×10^-3^ |
| rh_superiorparietal_part1 | -3.19 | 1.76×10^-3^ |
| rh_supramarginal_part2 | 3.48 | 6.70×10^-4^ |
| rh_supramarginal_part4 | 5.06 | 1.25×10^-6^ |
| rh_supramarginal_part5 | 3.41 | 8.43×10^-4^ |
| rh_Thalamus | -5.67 | 7.77×10^-8^ |
| rh_Putamen | -2.88 | 4.62×10^-3^ |
| rh_Amygdala | 3.73 | 2.78×10^-4^ |

^a^t-statistics were calculated by general linear model after regressing out age, sex, and TIV.

^b^All P values were corrected by BH-FDR.

lh, left hemisphere; rh, right hemisphere; BH-FDR, Benjamini-Hochberg false discovery rate; FBTCS+, with focal to bilateral tonic-clonic seizures.

Supplementary Table 3: Statistically significant regional difference between FBTCS- and HC.

| Regions | t-statistic^a^ | P_FDR_^b^ |
| --- | --- | --- |
| lh_inferiorparietal_part3 | 3.23 | 1.74×10^-3^ |
| lh_inferiortemporal_part6 | -3.53 | 6.60×10^-4^ |
| lh_lateraloccipital_part8 | -3.34 | 1.23×10^-3^ |
| lh_parahippocampal_part2 | -3.70 | 3.67×10^-4^ |
| lh_precentral_part3 | -4.57 | 1.53×10^-5^ |
| lh_precuneus_part2 | 3.62 | 4.87×10^-4^ |
| lh_precuneus_part3 | 3.50 | 7.26×10^-4^ |
| lh_superiortemporal_part1 | -3.28 | 1.47×10^-3^ |
| lh_Thalamus | -4.86 | 4.83×10^-6^ |
| lh_Putamen | -3.60 | 5.11×10^-4^ |
| lh_Pallidum | -3.58 | 5.54×10^-4^ |
| lh_Hippocampus | -9.56 | 2.13×10^-15^ |
| rh_fusiform_part3 | 3.82 | 2.43×10^-4^ |
| rh_inferiorparietal_part5 | -3.39 | 1.02×10^-3^ |
| rh_lateralorbitofrontal_part1 | 3.50 | 7.33×10^-4^ |
| rh_superiorparietal_part7 | 3.25 | 1.60×10^-3^ |
| rh_supramarginal_part2 | 3.61 | 5.08×10^-4^ |
| rh_supramarginal_part4 | 5.12 | 1.69×10^-6^ |
| rh_Thalamus | -3.95 | 1.56×10^-4^ |

^a^t-statistics were calculated by general linear model after regressing out age, sex, and TIV.

^b^All P values were corrected by BH-FDR.

lh, left hemisphere; rh, right hemisphere; BH-FDR, Benjamini-Hochberg false discovery rate; FBTCS-, without focal to bilateral tonic-clonic seizures.

Supplementary Table 4: The difference of GMV in each Yeo network between TLE and HC.

| Networks | VN | SMN | DAN | VAN | LN | FPN | DMN |
| --- | --- | --- | --- | --- | --- | --- | --- |
| *t*-statistic^a^ | -0.533 | -2.800 | -1.438 | -0.319 | 0.631 | -1.640 | -1.288 |
| P_FDR_^b^ | 0.694 | 0.040* | 0.349 | 0.750 | 0.694 | 0.349 | 0.349 |

^a^*t*-statistics were calculated by general linear model after regressing out age, sex, and TIV.

^b^All P values were corrected by BH-FDR

^*^ indicated P_FDR_<0.05

BH-FDR, Benjamini-Hochberg false discovery rate; DAN, dorsal attention network; DMN, default mode network; FPN, fronto-parietal network; GMV, gray matter volume; HC, healthy controls; LN limbic network; SMN, somato-motor network; TLE, temporal lobe epilepsy; VAN, ventral attention network, VN, visual network.

Supplementary Table 5: The difference of GMV in each von Economo class between TLE and HC.

| Networks | Prim motor | Asso1 | Asso2 | Sec sens | Prim sense | Limbic | Insula |
| --- | --- | --- | --- | --- | --- | --- | --- |
| *t*-statistic^a^ | -2.775 | -1.112 | -1.640 | -0.087 | 0.159 | -1.120 | -0.209 |
| P_FDR_^b^ | 0.043* | 0.468 | 0.360 | 0.931 | 0.931 | 0.468 | 0.931 |

^a^*t*-statistics were calculated by general linear model after regressing out age, sex, and TIV.

^b^All P values were corrected by BH-FDR

^*^ indicated P_FDR_<0.05

Asso1, association cortex 1; Asso2, association cortex 2; BH-FDR, Benjamini-Hochberg false discovery rate; GMV, gray matter volume; HC, healthy controls; Prim motor, primary motor cortex; Prim sens, primary sensory cortex; Sec sens, second sensory cortex, TLE, temporal lobe epilepsy.

Supplementary Table 6: The difference of GMV in each Yeo network between FBTCS- and HC.

| Networks | VN | SMN | DAN | VAN | LN | FPN | DMN |
| --- | --- | --- | --- | --- | --- | --- | --- |
| *t*-statistic^a^ | -0.911 | -1.512 | -0.371 | 1.290 | 0.621 | 0.151 | -0.226 |
| P_FDR_^b^ | 0.852 | 0.701 | 0.880 | 0.701 | 0.880 | 0.880 | 0.880 |

^a^*t*-statistics were calculated by general linear model after regressing out age, sex, and TIV.

^b^All P values were corrected by BH-FDR

BH-FDR, Benjamini-Hochberg false discovery rate; DAN, dorsal attention network; DMN, default mode network; FPN, fronto-parietal network; FBTCS-, without focal to bilateral tonic-clonic seizures; GMV, gray matter volume; HC, healthy controls; LN limbic network; SMN, somato-motor network; VAN, ventral attention network, VN, visual network.

Supplementary Table 7: The difference of GMV in each von Economo class between FBTCS- and HC.

| Networks | Prim motor | Asso1 | Asso2 | Sec sens | Prim sense | Limbic | Insula |
| --- | --- | --- | --- | --- | --- | --- | --- |
| *t*-statistic^a^ | -2.027 | 0.032 | 0.126 | -0.558 | 0.402 | -0.669 | 1.279 |
| P_FDR_^b^ | 0.319 | 0.975 | 0.975 | 0.964 | 0.964 | 0.964 | 0.715 |

^a^*t*-statistics were calculated by general linear model after regressing out age, sex, and TIV.

^b^All P values were corrected by BH-FDR

Asso1, association cortex 1; Asso2, association cortex 2; BH-FDR, Benjamini-Hochberg false discovery rate; FBTCS-, without focal to bilateral tonic-clonic seizures; GMV, gray matter volume; HC, healthy controls; Prim motor, primary motor cortex; Prim sens, primary sensory cortex; Sec sens, second sensory cortex.

Supplementary Table 8: The difference of GMV in each Yeo network between FBTCS+ and HC.

| Networks | VN | SMN | DAN | VAN | LN | FPN | DMN |
| --- | --- | --- | --- | --- | --- | --- | --- |
| *t*-statistic^a^ | -0.245 | -2.876 | -1.651 | -0.984 | 0.493 | -2.193 | -1.562 |
| P_FDR_^b^ | 0.806 | 0.032^*^ | 0.211 | 0.457 | 0.727 | 0.105 | 0.211 |

^a^*t*-statistics were calculated by general linear model after regressing out age, sex, and TIV.

^b^All P values were corrected by BH-FDR

^*^ indicated P_FDR_<0.05

BH-FDR, Benjamini-Hochberg false discovery rate; DAN, dorsal attention network; DMN, default mode network; FPN, fronto-parietal network; FBTCS+, with focal to bilateral tonic-clonic seizures; GMV, gray matter volume; HC, healthy controls; LN limbic network; SMN, somato-motor network; VAN, ventral attention network, VN, visual network.

Supplementary Table 9: The difference of GMV in each von Economo class between FBTCS+ and HC.

| Networks | Prim motor | Asso1 | Asso2 | Sec sens | Prim sense | Limbic | Insula |
| --- | --- | --- | --- | --- | --- | --- | --- |
| *t*-statistic^a^ | -2.502 | -1.383 | -2.320 | 0.117 | 0.082 | -1.049 | -0.934 |
| P_FDR_^b^ | 0.076 | 0.394 | 0.076 | 0.935 | 0.935 | 0.493 | 0.493 |

^a^*t*-statistics were calculated by general linear model after regressing out age, sex, and TIV.

^b^All P values were corrected by BH-FDR

Asso1, association cortex 1; Asso2, association cortex 2; BH-FDR, Benjamini-Hochberg false discovery rate; FBTCS+, with focal to bilateral tonic-clonic seizures; GMV, gray matter volume; HC, healthy controls; Prim motor, primary motor cortex; Prim sens, primary sensory cortex; Sec sens, second sensory cortex.

Supplementary Table 10: The relationship between clinical factors and statistically significant regional GMV in FBTCS-.

| Regions | Seizure onset age | | Seizure frequency | |
| --- | --- | --- | --- | --- |
|  | r^a^ | P_FDR_ | t-statistic^b^ | P_FDR_ |
| lh_inferiorparietal_part3 | 0.102 | 0.750 | -2.470 | 0.182 |
| lh_inferiortemporal_part6 | 0.251 | 0.750 | -0.780 | 0.699 |
| lh_lateraloccipital_part8 | 0.087 | 0.750 | 0.491 | 0.794 |
| lh_parahippocampal_part2 | -0.071 | 0.872 | 0.696 | 0.718 |
| lh_precentral_part3 | -0.062 | 0.872 | -0.094 | 0.926 |
| lh_precuneus_part2 | 0.145 | 0.750 | -1.889 | 0.324 |
| lh_precuneus_part3 | 0.216 | 0.750 | -1.204 | 0.564 |
| lh_superiortemporal_part1 | 0.165 | 0.750 | -1.736 | 0.352 |
| lh_Thalamus | 0.023 | 0.750 | -0.872 | 0.674 |
| lh_Putamen | -0.257 | 0.750 | 0.111 | 0.926 |
| lh_Pallidum | -0.141 | 0.750 | -0.428 | 0.797 |
| lh_Hippocampus | 0.142 | 0.554 | -1.442 | 0.432 |
| rh_fusiform_part3 | -0.114 | 0.750 | -2.608 | 0.182 |
| rh_inferiorparietal_part5 | -0.216 | 0.750 | 1.510 | 0.432 |
| rh_lateralorbitofrontal_part1 | -0.127 | 0.750 | -1.991 | 0.324 |
| rh_superiorparietal_part7 | -0.016 | 0.750 | 0.537 | 0.794 |
| rh_supramarginal_part2 | 0.105 | 0.872 | 0.933 | 0.674 |
| rh_supramarginal_part4 | 0.211 | 0.750 | 0.369 | 0.799 |
| rh_Thalamus | -0.153 | 0.765 | -1.129 | 0.565 |

^a^r values were calculated by Spearman correlation between clinical factors and statistically significant regional GMV.

^b^t-statistics were calculated by general linear model after regressing out age, sex, and TIV.

All P values were corrected by BH-FDR.

lh, left hemisphere; rh, right hemisphere; BH-FDR, Benjamini-Hochberg false discovery rate; FBTCS-, without focal to bilateral tonic-clonic seizures.

Supplementary Table 11: The relationship between clinical factors and statistically significant regional GMV in FBTCS+.

| Regions | Seizure onset age | | Seizure frequency | |
| --- | --- | --- | --- | --- |
|  | r^a^ | P_FDR_ | t-statistic^b^ | P_FDR_ |
| lh_cuneus_part1 | -0.084 | 0.849 | 0.072 | 0.988 |
| lh_inferiortemporal_part6 | 0.056 | 0.849 | -1.254 | 0.741 |
| lh_middletemporal_part4 | 0.085 | 0.849 | -0.313 | 0.988 |
| lh_middletemporal_part5 | 0.088 | 0.849 | -0.351 | 0.988 |
| lh_parahippocampal_part2 | 0.222 | 0.292 | -0.124 | 0.988 |
| lh_pericalcarine_part2 | 0.049 | 0.849 | 0.597 | 0.890 |
| lh_postcentral_part4 | -0.106 | 0.849 | -0.176 | 0.988 |
| lh_precentral_part3 | 0.048 | 0.849 | 0.590 | 0.890 |
| lh_precuneus_part1 | -0.100 | 0.849 | 0.533 | 0.909 |
| lh_precuneus_part2 | -0.044 | 0.849 | -0.648 | 0.890 |
| lh_precuneus_part3 | -0.023 | 0.877 | -0.906 | 0.817 |
| lh_precuneus_part7 | -0.090 | 0.849 | -1.011 | 0.741 |
| lh_superiorparietal_part3 | 0.073 | 0.849 | 0.025 | 0.988 |
| lh_superiorparietal_part6 | 0.037 | 0.849 | -0.049 | 0.988 |
| lh_superiortemporal_part1 | 0.378 | 0.011^*^ | -0.762 | 0.890 |
| lh_superiortemporal_part3 | 0.298 | 0.093 | -0.143 | 0.988 |
| lh_supramarginal_part2 | -0.108 | 0.849 | -1.011 | 0.741 |
| lh_supramarginal_part3 | -0.050 | 0.849 | 1.188 | 0.741 |
| lh_supramarginal_part4 | 0.102 | 0.849 | -1.023 | 0.741 |
| lh_supramarginal_part5 | -0.006 | 0.976 | -1.020 | 0.741 |
| lh_supramarginal_part6 | 0.064 | 0.849 | -1.171 | 0.741 |
| lh_insula_part3 | 0.067 | 0.849 | -0.646 | 0.890 |
| lh_Thalamus | 0.261 | 0.136 | -1.886 | 0.741 |
| lh_Caudate | -0.034 | 0.849 | 1.550 | 0.741 |
| lh_Putamen | 0.144 | 0.849 | -0.724 | 0.890 |
| lh_Pallidum | 0.132 | 0.849 | 0.139 | 0.988 |
| lh_Hippocampus | 0.263 | 0.136 | -3.098 | 0.106 |
| rh_bankssts_part2 | -0.097 | 0.849 | 0.215 | 0.988 |
| rh_fusiform_part3 | -0.023 | 0.877 | -1.633 | 0.741 |
| rh_inferiorparietal_part3 | 0.003 | 0.976 | -1.055 | 0.741 |
| rh_inferiorparietal_part7 | 0.040 | 0.849 | -0.015 | 0.988 |
| rh_rostralanteriorcingulate_part1 | -0.050 | 0.849 | -0.224 | 0.988 |
| rh_superiorfrontal_part5 | -0.144 | 0.849 | -1.610 | 0.741 |
| rh_superiorparietal_part1 | -0.110 | 0.849 | -1.269 | 0.741 |
| rh_supramarginal_part2 | 0.038 | 0.849 | -0.599 | 0.890 |
| rh_supramarginal_part4 | 0.051 | 0.849 | -1.022 | 0.741 |
| rh_supramarginal_part5 | -0.032 | 0.849 | -0.133 | 0.988 |
| rh_Thalamus | 0.064 | 0.849 | -1.326 | 0.741 |
| rh_Putamen | 0.160 | 0.849 | -0.507 | 0.909 |
| rh_Amygdala | -0.041 | 0.849 | -2.500 | 0.288 |

^a^r values were calculated by Spearman correlation between clinical factors and statistically significant regional GMV.

^b^t-statistics were calculated by general linear model after regressing out age, sex, and TIV.

All P values were corrected by BH-FDR.

^*^ indicated P_FDR_<0.05

lh, left hemisphere; rh, right hemisphere; BH-FDR, Benjamini-Hochberg false discovery rate; FBTCS+, with focal to bilateral tonic-clonic seizures.

Supplementary Table 12: Enrichment analysis of PLS+ genes with TLE related genes and other brain disorders related genes.

|  | FBTCS- | | FBTCS+ | |
| --- | --- | --- | --- | --- |
|  | ER | P_FDR_ | ER | P_FDR_ |
| TLE down regulated | 6.76 | 0.001^*^ | 7.69 | 0.001^*^ |
| TLE up regulated | -1.60 | 0.991 | -1.67 | 0.983 |
| ASD down regulated | 2.70 | 0.007^*^ | 2.97 | 0.019^*^ |
| ASD up regulated | -0.07 | 0.991 | -1.61 | 0.983 |
| SCZ down regulated | 1.37 | 0.339 | 2.06 | 0.084 |
| SCZ up regulated | -0.88 | 0.991 | -0.58 | 0.943 |
| BD down regulated | 0.92 | 0.479 | 0.97 | 0.511 |
| BD up regulated | -0.13 | 0.991 | -1.67 | 0.983 |
| MDD down regulated | -2.11 | 0.991 | -0.28 | 0.943 |
| MDD up regulated | -0.94 | 0.991 | -0.26 | 0.943 |
| AAD down regulated | -1.24 | 0.991 | -0.54 | 0.943 |
| AAD up regulated | NA | NA | NA | NA |

All P values were corrected by BH-FDR.

^*^ indicated P_FDR_<0.05;

AAD, alcohol abuse or dependence; ASD, autism spectrum disorder; BD, bipolar disorder, BH-FDR Benjamini–Hochberg false discovery rate; ER: enrichment ratio; FBTCS, focal to bilateral tonic-clonic seizures; MDD, major depressive disorder; SCZ, schizophrenia.

Supplementary Table 13: Enrichment analysis of PLS- genes with TLE related genes and other brain disorders related genes.

|  | FBTCS- | | FBTCS+ | |
| --- | --- | --- | --- | --- |
|  | ER | P_FDR_ | ER | P_FDR_ |
| TLE down regulated | -0.40 | 0.943 | -1.51 | 0.940 |
| TLE up regulated | 1.51 | 0.314 | 3.97 | 0.001^*^ |
| ASD down regulated | -1.29 | 0.943 | -1.33 | 0.940 |
| ASD up regulated | 2.00 | 0.314 | 2.62 | 0.018^*^ |
| SCZ down regulated | -1.50 | 0.943 | -1.24 | 0.940 |
| SCZ up regulated | 0.70 | 0.608 | 2.02 | 0.049^*^ |
| BD down regulated | -1.08 | 0.943 | 0.13 | 0.669 |
| BD up regulated | 1.05 | 0.559 | 0.44 | 0.611 |
| MDD down regulated | 0.00 | 0.943 | 2.27 | 0.049^*^ |
| MDD up regulated | -0.86 | 0.943 | -0.68 | 0.940 |
| AAD down regulated | -0.65 | 0.943 | 0.50 | 0.574 |
| AAD up regulated | NA | NA | NA | NA |

All P values were corrected by BH-FDR.

^*^ indicated P_FDR_<0.05;

AAD, alcohol abuse or dependence; ASD, autism spectrum disorder; BD, bipolar disorder, BH-FDR Benjamini–Hochberg false discovery rate; ER: enrichment ratio; FBTCS, focal to bilateral tonic-clonic seizures; MDD, major depressive disorder; SCZ, schizophrenia.

Supplementary Table 14: Enrichment analysis of PLS+ genes for each cell type in FBTCS- and FBTCS+ groups.

| Cell types | FBTCS- | | | FBTCS+ | | |
| --- | --- | --- | --- | --- | --- | --- |
|  | ER | P_FDR_ | -log_10_P_FDR_ | ER | P_FDR_ | -log_10_P_FDR_ |
| Astrocytes | 0.01 | 0.664 | 0.178 | 1.02 | 0.300 | 0.523 |
| Endothelial cells | -0.74 | 0.858 | 0.067 | -1.04 | 0.855 | 0.068 |
| Microglia | 1.57 | 0.166 | 0.781 | 1.69 | 0.126 | 0.900 |
| Excitatory neurons | 5.91 | 0.001^*^ | 3.155 | 4.39 | 0.001^*^ | 3.155 |
| Inhibitory neurons | 2.77 | 0.009^*^ | 2.025 | 1.99 | 0.123 | 0.911 |
| oligodendrocytes | -1.37 | 0.915 | 0.039 | -0.78 | 0.855 | 0.068 |
| OPCs | 0.53 | 0.492 | 0.308 | -0.58 | 0.855 | 0.068 |

All P values were corrected by BH-FDR.

^*^ indicated P_FDR_<0.05;

BH-FDR Benjamini–Hochberg false discovery rate; FBTCS, focal to bilateral tonic-clonic seizures; OPCs: oligodendrocyte precursor cells.

Supplementary Table 15: Statistically significant regional difference between current-FBTCS+ and HC.

| Regions | t-statistic^a^ | P_FDR_^b^ |
| --- | --- | --- |
| lh_cuneus_part1 | 3.29 | 1.32×10^-3^ |
| lh_inferiortemporal_part6 | -3.24 | 1.55×10^-3^ |
| lh_middletemporal_part4 | -3.35 | 1.10×10^-3^ |
| lh_middletemporal_part5 | -3.75 | 2.82×10^-4^ |
| lh_superiorfrontal_part6 | -3.05 | 2.88×10^-3^ |
| lh_superiortemporal_part1 | -4.84 | 4.12×10^-6^ |
| lh_superiortemporal_part3 | -3.57 | 5.18×10^-4^ |
| lh_supramarginal_part1 | -2.95 | 3.86×10^-3^ |
| lh_supramarginal_part2 | -3.08 | 2.57×10^-3^ |
| lh_supramarginal_part3 | -4.11 | 7.40×10^-5^ |
| lh_supramarginal_part4 | -2.93 | 4.12×10^-3^ |
| lh_supramarginal_part5 | -3.71 | 3.23×10^-4^ |
| lh_supramarginal_part6 | -3.51 | 6.48×10^-4^ |
| lh_insula_part3 | -4.01 | 1.10×10^-4^ |
| lh_thalamus | -5.02 | 1.97×10^-6^ |
| lh_caudate | -3.24 | 1.57×10^-3^ |
| lh_putamen | -3.94 | 1.41×10^-4^ |
| lh_pallidum | -3.74 | 2.92×10^-4^ |
| lh_hippocampus | -8.81 | 1.74×10^-14^ |
| rh_fusiform_part3 | 3.77 | 2.59×10^-4^ |
| rh_inferiorparietal_part3 | -3.18 | 1.88×10^-3^ |
| rh_rostralanteriorcingulate_part1 | 3.64 | 4.11×10^-4^ |
| rh_superiorfrontal_part5 | -3.31 | 1.25×10^-3^ |
| rh_superiorparietal_part1 | -3.08 | 2.56×10^-3^ |
| rh_supramarginal_part2 | 3.29 | 1.35×10^-3^ |
| rh_supramarginal_part4 | 4.59 | 1.14×10^-5^ |
| rh_supramarginal_part5 | 2.93 | 4.16×10^-3^ |
| rh_thalamus | -5.22 | 8.19×10^-7^ |
| rh_amygdala | 3.66 | 3.91×10^-4^ |

^a^t-statistics were calculated by general linear model after regressing out age, sex, and TIV.

^b^All P values were corrected by BH-FDR.

lh, left hemisphere; rh, right hemisphere; BH-FDR, Benjamini-Hochberg false discovery rate; FBTCS+, with focal to bilateral tonic-clonic seizures.

Supplementary Table 16: Statistically significant regional difference between remote-FBTCS+ and HC.

| Regions | t-statistic^a^ | P_FDR_^b^ |
| --- | --- | --- |
| lh_cuneus_part1 | 3.67 | 4.31×10^-4^ |
| lh_inferiortemporal_part1 | 4.17 | 7.46×10^-5^ |
| lh_inferiortemporal_part2 | 3.62 | 5.03×10^-4^ |
| lh_parahippocampal_part2 | -3.80 | 2.71×10^-4^ |
| lh_precentral_part3 | -3.35 | 1.19×10^-3^ |
| lh_superiorparietal_part3 | -3.39 | 1.05×10^-3^ |
| lh_superiortemporal_part1 | -4.44 | 2.69×10^-5^ |
| lh_supramarginal_part3 | -3.50 | 7.48×10^-4^ |
| lh_supramarginal_part5 | -3.81 | 2.65×10^-4^ |
| lh_insula_part3 | -3.12 | 2.44×10^-3^ |
| lh_thalamus | -4.49 | 2.24×10^-5^ |
| lh_putamen | -5.51 | 3.88×10^-7^ |
| lh_hippocampus | -9.64 | 3.11×10^-15^ |
| rh_fusiform_part3 | 3.91 | 1.85×10^-4^ |
| rh_supramarginal_part4 | 4.35 | 3.82×10^-5^ |
| rh_thalamus | -5.04 | 2.62×10^-6^ |
| rh_putamen | -3.10 | 2.63×10^-3^ |

^a^t-statistics were calculated by general linear model after regressing out age, sex, and TIV.

^b^All P values were corrected by BH-FDR.

lh, left hemisphere; rh, right hemisphere; BH-FDR, Benjamini-Hochberg false discovery rate; FBTCS+, with focal to bilateral tonic-clonic seizures.

Supplementary Table 17: Enrichment analysis for each cell type in current-FBTCS+ and remote-FBTCS+ groups.

| Cell types | current-FBTCS+ | | | remote-FBTCS+ | | |
| --- | --- | --- | --- | --- | --- | --- |
|  | ER | P_FDR_ | -log_10_P_FDR_ | ER | P_FDR_ | -log_10_P_FDR_ |
| Astrocytes | 0.24 | 0.703 | 0.153 | 0.36 | 0.589 | 0.230 |
| Endothelial cells | -0.91 | 0.876 | 0.058 | 0.03 | 0.589 | 0.230 |
| Microglia | 0.88 | 0.540 | 0.267 | 0.12 | 0.589 | 0.230 |
| Excitatory neurons | 3.79 | 0.001^*^ | 3.155 | 6.02 | 0.001^*^ | 3.155 |
| Inhibitory neurons | 0.81 | 0.540 | 0.267 | 2.66 | 0.011^*^ | 1.951 |
| oligodendrocytes | -1.14 | 0.876 | 0.058 | -1.59 | 0.944 | 0.025 |
| OPCs | -0.53 | 0.876 | 0.058 | 0.11 | 0.589 | 0.230 |

All P values were corrected by BH-FDR.

^*^ indicated P_FDR_<0.05;

BH-FDR Benjamini–Hochberg false discovery rate; OPCs: oligodendrocyte precursor cells. FBTCS+, with focal to bilateral tonic-clonic seizures.


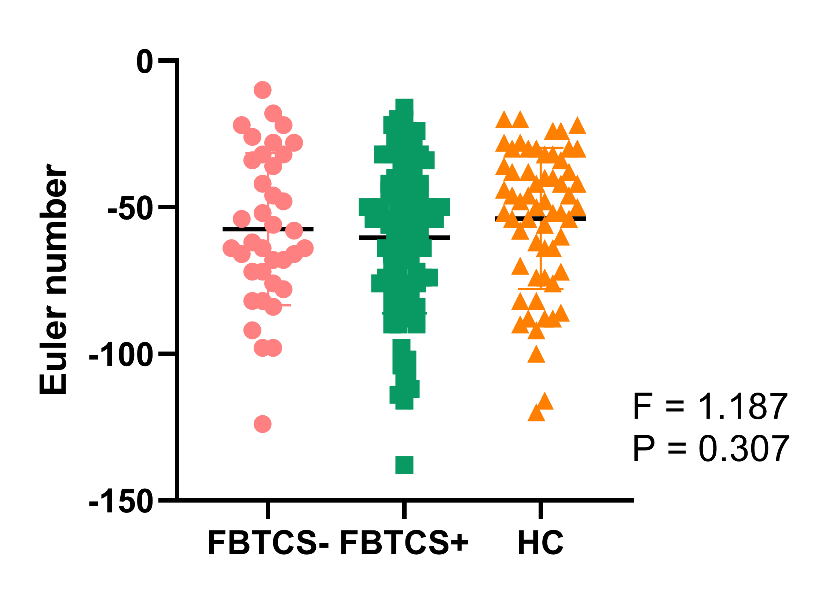


Figure S1: Euler number comparison among FBTCS-, FBTCS+ and HC.


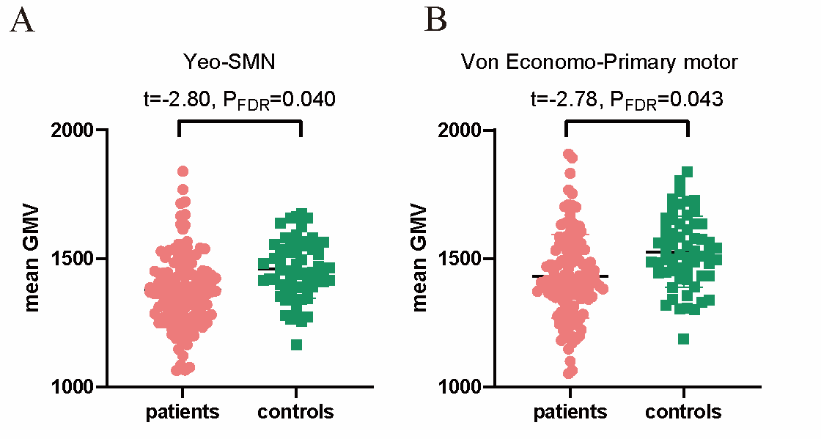


**Figure S2: Statistically significant differences of GMV within Yeo network and von Economo classes in TLE patients.** (A) The difference of GMV in SMN between TLE patients and controls. (B) (A) GMV differences in primary motor cortex between groups. SMN, somato-motor network.


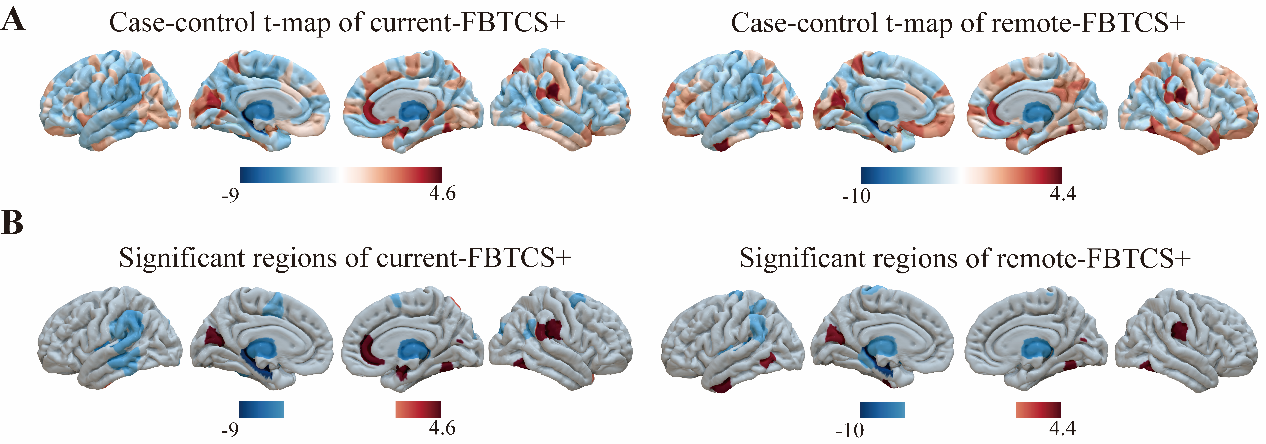


Figure S3: The differences of GMV in current-FBTCS+ and remote-FBTCS+ compared with HC. (A) Case-control t-map of regional GMV in current-FBTCS+ (left), remote-FBTCS+ (right) vs. HC. (B) Statistical significant regions in current-FBTCS+ (left), remote-FBTCS+ (right) vs. HC. after FDR correction. FBTCS-, without focal to bilateral tonic-clonic seizures; FBTCS+, with focal to bilateral tonic-clonic seizures; GMV, gray matter volume.


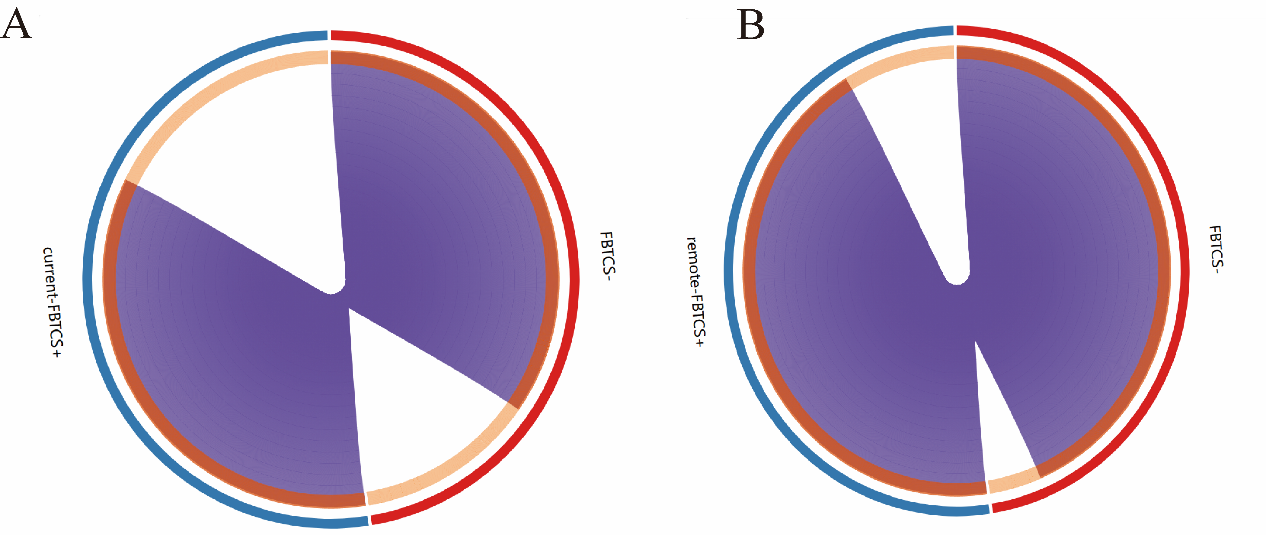


Figure S4: The Circos map displayed overlapped genes. (A) The overlapped genes between FBTCS- and current-FBTCS+; (B) The overlapped genes between FBTCS- and remote-FBTCS+. FBTCS-, without focal to bilateral tonic-clonic seizures; FBTCS+, with focal to bilateral tonic-clonic seizures.


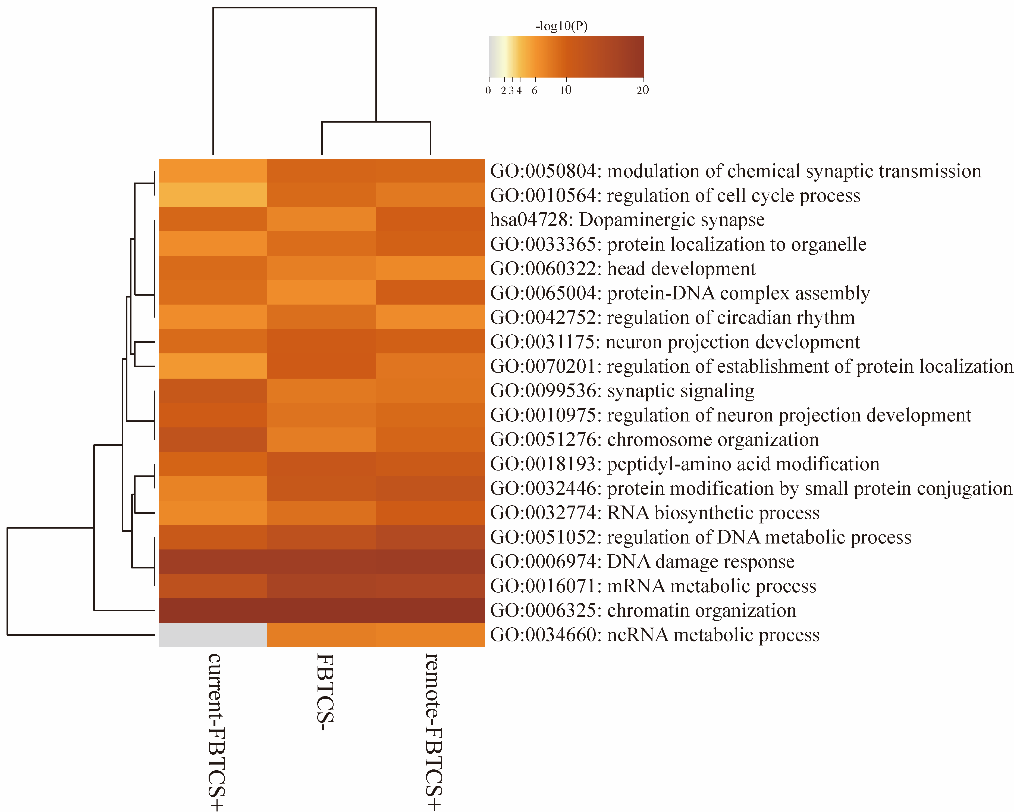


Figure S5: The heatmap displayed the GO and KEGG pathways for multi-gene-list of current-FBTCS+, remote-FBTCS+ and FBTCS-. FBTCS-, without focal to bilateral tonic-clonic seizures; FBTCS+, with focal to bilateral tonic-clonic seizures.


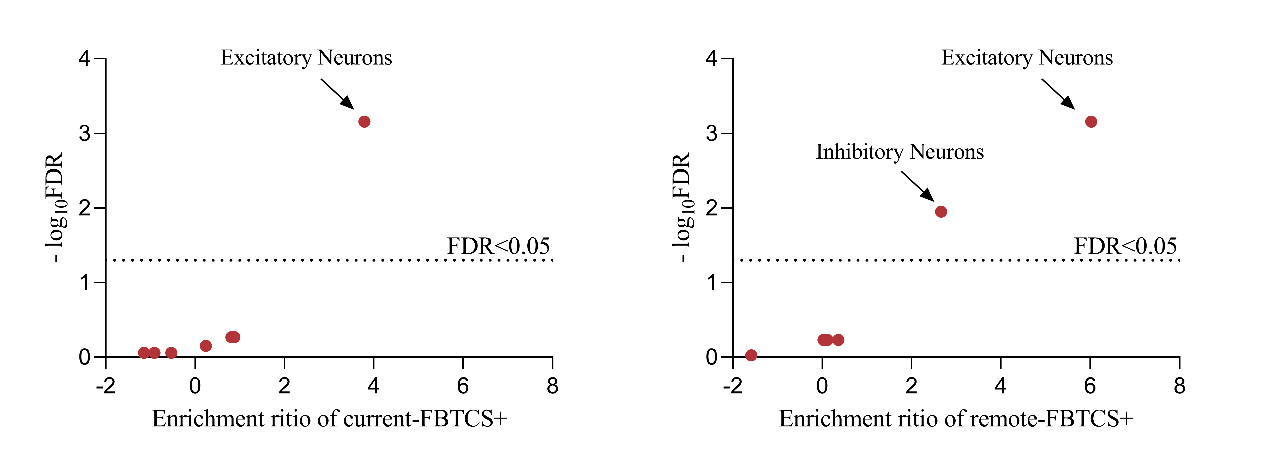


Figure S6: Cell type enrichment of PLS+ genes of current-FBTCS+ and remote-FBTCS+. FBTCS+, with focal to bilateral tonic-clonic seizures.
